# Change of cystine/glutamate antiporter expression in ethanol-dependent rats

**DOI:** 10.3389/fnins.2014.00311

**Published:** 2014-10-06

**Authors:** Alessandra T. Peana, Giulia Muggironi, Federico Bennardini

**Affiliations:** Department of Chemistry and Pharmacy, University of SassariSassari, Italy

**Keywords:** ethanol-dependent rats, ethanol non-dependent rats, withdrawal, cystine/glutamate antiporter

## Abstract

**Background:** Some drugs of abuse down regulate the expression of cystine/glutamate (xCT) antiporter in the nucleus accumbens (Acb) after extinction or withdrawal. The altered level of xCT exchanger in Acb, a structure involved in ethanol reinforcement, may contribute to the pathological glutamatergic signaling, linked to addiction. We hypothesized that the expression of xCT may be changed in Acb and whole brain also in non-dependent (occasional drinkers), ethanol-dependent rats, as well as, during ethanol withdrawal.

**Methods:** Wistar rats were made ethanol-dependent by chronic exposure to an alcoholic milk beverage (from 2.4 to 7.2% v/v ethanol). Ethanol non-dependent rats were exposed to a similar, but non-alcoholic liquid diet and self-administered ethanol (10%) twice a week. Withdrawal in ethanol-dependent rats was studied at 12 h after the last ethanol-enriched diet exposure. Immediately after the measurement of somatic signs of withdrawal, Western blot analysis with a polyclonal antibody against xCT was carried out in a naïve control group, non-dependent and ethanol-dependent rats as well as withdrawal rats, in order to study the level of xCT expression in Acb and whole brain.

**Results:** Non-dependent rats self-administered an average dose of 1.21 ± 0.02 g/kg per session (30 min). Daily ethanol consumption during chronic exposure to the alcoholic beverage ranged from 6.30 ± 0.16 to 13.99 ± 0.66 g/kg. Ethanol dependent rats after suspension of the ethanol-enriched diet have shown significant somatic signs of withdrawal. Western blotting analysis of Acb lysates revealed that xCT was over expressed in ethanol-dependent rats whereas in whole brain preparations xCT was over expressed in both non-dependent and ethanol-dependent rats compared to control group. On the contrary, xCT expression during withdrawal was down regulated in Acb and restored to control level in whole brain preparations.

**Conclusions:** The changes of xCT expression in both Acb and whole brain following ethanol dependence and withdrawal indicate that xCT might represent a novel therapeutic target for the treatment of ethanol addiction.

## Introduction

The development of ethanol dependence is posited to involve numerous changes in brain neurotransmission that lead to characteristic physiological signs upon abstinence from ethanol.

Increased glutamatergic neurotransmission appears to mediate the reinforcing properties of ethanol and changes are considered responsible of affecting many aspects of neuroplasticity associated with ethanol dependence (De Witte et al., 2005; Knackstedt and Kalivas, 2009; Bridges et al., 2012; Griffin Iii et al., 2014; Lum et al., 2014). The increase of extracellular glutamate caused by ethanol in many brain areas, could be related to the glutamate/cysteine exchanger presence in a wide variety of neuronal cells (Lewerenz et al., 2012). It is well known that the glutamate/cystine antiporter transports cystine into neuronal cells in exchange of glutamate at a ratio of 1:1. It is composed of a catalytic light chain subunit, xCT (also known as system xc- or Slc7a11), which mediates the ion co-transport, and a regulatory heavy chain subunit (4F2), linked by a disulfide bridge (Lewerenz et al., 2012). Internalized cystine is reduced into cysteine, the rate-limiting precursor of glutathione (GSH), while the externalized glutamate can contribute to either excitatory signaling or excitotoxicity (Seib et al., 2011). Particularly, drugs of abuse such as cocaine (Knackstedt et al., 2010) and nicotine (Knackstedt et al., 2009) produce a down-regulation of xCT antiporter, studied by Western blot analysis in different brain areas, either 3 weeks after cocaine extinction (Knackstedt et al., 2010) or 12 h after the last nicotine treatment (i.e., during withdrawal) (Knackstedt et al., 2009). This reduction in xCT system occurs, among other brain areas, in the Acb and may contribute to the pathological glutamate signaling linked to addiction (Bridges et al., 2012).

Recently, Pochareddy and Edenberg (2012) demonstrated that long-term ethanol exposure *in vitro* results in altered expression of roughly thousand genes in human hepatoma cells (HepG2) among which the *Slc7a11* gene, has been shown to be expressed at higher levels (1.54-fold), whereas other authors have shown that ethanol, dose-dependently, increases the xCT exchanger expression in mouse hepatic stellate cells (Lin et al., 2013). Notably, the main function of the xCT antiporter is to maintain the intracellular level of glutathione and protect cells from oxidative damage (Bannai, 1986; Pochareddy and Edenberg, 2012). Thus, up-regulation of xCT expression might be a compensatory mechanism in response to ethanol-induced oxidative stress. At present, little information is available concerning the role of xCT antiporter in the brain of both ethanol non-dependent (occasional drinkers) and ethanol-dependent rats as well as during withdrawal in ethanol-dependent rats. For this purpose, two groups were made dependent on ethanol by chronic exposure to an ethanol-containing liquid diet, prepared from cow's milk with some additions. Validation of the ethanol-containing liquid diet's ability to induce dependence (Uzbay and Kayaalp, 1995) was confirmed by the appearance of the ethanol withdrawal syndrome after ethanol suspension (Macey et al., 1996). Ethanol non-dependent rats (occasional drinkers) were exposed to a similar, but non-alcoholic liquid diet and were allowed to self-administer ethanol twice a week.

Western blot experiments were performed using both Acb and whole brain homogenates to determine if xCT expression was changed by the different experimental conditions.

## Materials and methods

The study was carried out in accordance with the current Italian legislation [D.L. 116, 1992], which allows experimentation on laboratory animals only after submission and approval of a research project to the Independent Committee of Bioethics of the University for Animal Testing (Sassari, Italy) and to the Ministry of Health (Rome, Italy), and in strict accordance with the European Council directives on the matter [n. 2007/526/CE]. All possible efforts were made to minimize animal pain and discomfort and to reduce the number of experimental subjects.

### Drugs and chemicals

Ethanol solutions (v/v) obtained by dilution of ethanol (95%; U.S. Pharmacopeia National Formulary, 1995) with tap water were freshly prepared before every session of self-administration. xCT polyclonal antibody was from Abnova (Taipei, Taiwan). β-tubulin antibody was purchased from Sigma-Aldrich (St. Louis, MO, USA). Both anti-mouse and anti-rabbit IgG-coupled horseradish peroxidase antibodies as well as enhanced chemiluminescence (ECL) reagents were from Cell Signaling Technology (USA). All other reagents were of the highest purity grade commercially available.

### Animals

Male Wistar rats (*n* = 58) (Harlan, Udine, Italy), weighing 175–225 g at the beginning of the experiment, were housed in pairs in Plexiglas cages. The colony room was maintained under controlled environmental conditions (temperature 22 ± 2°C; humidity 60–65%) under a 12-h light/dark cycle (light on at 8:00 h; off at 20:00 h).

Four groups of rats were utilized for this study:

1. Ethanol non-dependent rats allowed to self-administer ethanol twice a week (Monday and Thursday; *n* = 8). These rats were fed the non-alcoholic liquid diet.
2. Ethanol dependent rats (*n* = 27). These rats were fed the alcoholic liquid diet.
3. Ethanol dependent withdrawn: rats (*n* = 17) after suspension of the ethanol-enriched diet.
4. Control rats that was used only for xCT measurements, fed the non-alcoholic liquid diet (*n* = 6).

Both groups of rats, non-dependent and dependent have, in the home cage, a bottle of milk (liquid diet) with (dependent) or without ethanol (non-dependent). Non-dependent rats (occasional drinkers) drank for 30 min (twice a week) the “desired” amount of ethanol. Instead, the dependence was induced by “forced” ethanol liquid diet (method of Uzbay and Kayaalp, 1995).

### Liquid diet composition

The rats were given a modified liquid diet with (ethanol dependent) or without (control and ethanol non-dependent) ethanol *ad libitum*. No extra chow or water was supplied. The composition of the modified liquid diet with ethanol is:

fresh whole cow milk, 910–970 ml (CoaPla, Italy)/l;
ethanol 25–75 ml/l;
vitamin A 5000 IU/l
sucrose 17 g/l.

This mixture (with or without ethanol), freshly prepared daily, according to the method of Uzbay and Kayaalp (1995), supplies 1000.7 kcal/l. Briefly, the liquid diet without ethanol contains 17 g of sucrose instead, the liquid died with ethanol, at the time when ethanol concentration is increased, sucrose was reduced to maintain isocaloricity of the diet. The beverage (with or without ethanol) was presented at the same time of the day (09:30 h AM) for 24 h.

### Ethanol administration in non-dependent rats (occasional drinkers)

Training was conducted in modular operant chambers, located in ventilated soundproof environmental cubicles (Med Associates Inc. USA). Each chamber was equipped with a non-retractable drinking cup (capacity 0.50 ml) and two nose-poke holes located 3 cm to the left and right of the cup. A white light placed above the active hole and an orange light placed above the inactive hole were used as environmental stimuli. Only the active nose-poke hole set off the dipper-delivering solution (0.1 ml) into the drinking cup in 3.05-second period. Explorations at both the active and inactive nose-poke holes were recorded. In particular, recording at the inactive hole served to control for specificity of the response in the operant chamber. The availability of liquid was signaled by a house light placed on the wall in front of the drinking cup that would light up for the duration of liquid delivery. Following each delivery, there was a 2-second time-out period during which responses had no consequences and the white light placed above the active hole went off. An infrared head detector was located in the reservoir and recorded all signals during the entire session. The chambers were interfaced to a computer equipped with software that ran the programmed sessions and recorded the data. For operant ethanol (5–10% v/v) self-administration, rats were trained to nose-poke under a fixed-ratio 1 (FR1) schedule of reinforcement, in which each response resulted in 0.1 ml of solution delivery. From day 1 to day 6, rats were permitted to nose-poke explore for 5% ethanol solution. Starting on day 7, the ethanol percentage was gradually increased, with daily increases of 1% up to the final concentration of 10%. Following the acquisition, after a stable baseline of responding was reached, operant self-administration of ethanol was then increased to a FR2 schedule until the self-administration behavior was stable and subsequently, the schedule requirement was increased to a FR3 (Peana et al., 2013). After this period (approximately 30 days from acquisition training) the non-dependent rats (occasional drinkers) were allowed to self-administer ethanol twice a week (Monday and Thursday at 9.00 h).

### Ethanol administration in dependent rats

At the beginning of the study, the rats were given the liquid diet without ethanol for 7 days. Then, liquid diet, for inducing ethanol dependence, was gradually enriched with 2.4% (3 days), 4.8% (4 days) and 7.2% (14 days) ethanol. This diet composition and regimen has been reported to result in a significant correlation between ethanol-containing liquid diet consumption and blood ethanol level after 21 days of treatment, as reported by Kayir and Uzbay (2008). Rat's body weight (g), liquid intake (ml/kg) as well as ethanol intake (g/kg) were recorded daily.

### Observation of somatic signs of withdrawal

Withdrawal in ethanol-dependent rats was studied at 12 h after the last ethanol-enriched diet exposure. Withdrawal behavioral signs were determined exactly at 12 h after ethanol suspension. Each subject was placed under white light conditions in Plexiglas observation chambers (25 × 20 × 25 cm) and observed for 5 min by an observer blind to the subject's treatments. The following somatic signs of withdrawal were recorded: body tremors (BT), tail rigidity (TR), vocalization (VOC) and ventro-medial limb retraction (VmLR). We used a rating scale adapted from Macey et al. (1996) as follows: 0 = no sign, 1 = moderate, 2 = severe.

To measure anxiety-like responses upon ethanol withdrawal, the elevated plus maze (EPM) test was used. The test was performed immediately, after 12 h after ethanol suspension. The apparatus consisted of 2 gray Plexiglas open arms and 2 black enclosed arms (40-cm high walls), with similarly shaped arms opposite to each other. The 5-min test procedure began when the animal was placed in the center of the maze, facing an open arm. The time (min) spent in open arms and the number of open arm entries were scored and used as measure of anxiety-like behavior (Cruz et al., 1994).

All behavioral testing for ethanol non-dependent and for dependent rats were determined at the same time corresponding exactly at 12 h after ethanol suspension in abstinent rats. All experiments were carried out during the light period in a room with a soft light.

### Cystine/glutamate antiporter expression

Immediately after the behavioral observations, rats were intra-peritoneally injected with 1.3 g/kg of ethylic urethane (Sigma-Aldrich, Milan, Italy). Under deep anesthesia, rats were sacrificed and brains removed and immediately frozen at −80°C (4 h) before being sectioned. Brains were rapidly dissected into coronal sections on an ice-cooled metal plate using a scalpel. The brain regions were identified according to the rat brain atlas (Paxinos and Watson, 1998) and from these slices two sections (approximately 2 mm thick), containing Acb, were isolated and identified by visual inspection and direct comparison with the images of the rat brain atlas in stereotaxic coordinates corresponding to AP +1.7 to +1.9 mm from bregma (Paxinos and Watson, 1998). The bilateral brain sites containing Acb (shell an core; control group: *n* = 3; ethanol non-dependent: *n* = 5; ethanol dependent: *n* = 7; withdrawal in ethanol dependent: *n* = 7) and whole brain sections (control group: *n* = 3; ethanol non-dependent: *n* = 3; ethanol dependent: *n* = 3; withdrawal in ethanol dependent: *n* = 3) were suspended in saline and subsequently homogenized as described below.

### Brain and nucleus accumbens homogenates preparation

Acb-containing slice portions or whole brains including Acb were weighted and homogenized in 1 ml and 4 ml, respectively, of ice-cold lysis buffer (20 mM Tris–HCl, pH 7.5, containing 150 mM NaCl, 1 mM EDTA, 1 mM EGTA, 1% Triton X-100, 1 mM phenylmethylsulfonylfluoride (PMSF), 1 mM β-glycerophosphate, 2.5 mM sodium pyrophosphate, 1 mM Na_3_VO_4_ and 1 μg/ml leupeptin). Homogenization was carried out with a Dounce homogenizer using 20 strokes of the loosely fitting pestle. The lysates were sonicated with an ultrasonic homogenizer (BioLogics, USA) for 20 s on ice at 20% amplitude, and centrifuged at 12,000 × g for 15 min at 4°C. The protein content of supernatants was measured according to Bradford (1976), using bovine serum albumin (BSA) as a standard. Lysates were used immediately for sodium dodecyl sulfate-polyacrylamide gel electrophoresis (SDS-PAGE) and Western blot analysis or stored at −20°C until future analysis.

### Western blot

Aliquots of protein lysates (80 μg) were separated on 12% SDS gel (Laemmli, 1970) and transferred to nitrocellulose membranes at 250 mA (constant current) for 1 h according to Towbin et al. (1979). After transfer the blot was saturated in TBS (20 mM Tris-HCl, pH 7.0, 150 mM NaCl), containing 5% non-fat milk powder (MP) and 0.05% Tween 20 for 1 h at room temperature (RT). The immunoreaction was carried out in TBS, 5% MP, 0.05% Tween 20, containing the Slc7a11 (xCT) polyclonal antibody diluted 1:1000 overnight at 4°C. After being washed three times with TBS, 5% MP, 0.05% Tween 20 (5 min each), the blot was incubated with goat anti-rabbit Ig-coupled horseradish peroxidase diluted 1:2000 in TBS, 5% MP, 0.05% Tween 20 for 1 h at RT. The nitrocellulose sheet was rinsed three times in TBS, 5% MP, 0.05% Tween 20 (5 min each) and once in water (10 min), before developing the reaction by ECL. After extensive washing with TBS, the bzlot was reprobed for the presence of β-tubulin with a monoclonal antibody diluted 1:1000 in TBS, 5% MP, 0.05% Tween 20 for 1 h at RT. Results were recorded on X-ray film (Kodak, USA) and analyzed by densitometric scanning using the ImageJ 1.47v open source public domain software developed at the National Institutes of Health, USA (http://imagej.nih.gov/ij/).

### Statistical analysis

All values are expressed as mean (± s.e.m.). Ethanol intake values (in dependent rats) were analyzed by one-way ANOVA. Rat body weights were analyzed by two-way ANOVA (repeated measure). The 4 different withdrawal signs (VOC, TR, BT and VmLR) were assessed by individual comparison among individual means using the non-parametric Mann-Whitney *U*-test. Numbers of open arm entries were analyzed by two-way ANOVA. Time spent in open arms were analyzed by one-way ANOVA. In the presence of overall significant main effects and interactions (*p*-values < 0.05), the Least Significant Differences (LSD) *post-hoc* test was performed.

Following densitometric analysis of the Western blot autoradiograms, data were expressed as arbitrary units (A.U.) of 3 different experiments ± s.e.m. and analyzed by unpaired Student's *t* test, assuming a *p* < 0.05 as statistically significant. A power analysis for *t*-test with the free software G^*^Power 3.1 (http://www.gpower.hhu.de/) was also carried out. The results of this analysis gave a power (1-β err *prob* = 0.95) with a sample size of 3 and an α level of 0.05. Being the power higher than 0.80 (Faul et al., 2007) there was no need to increase the sample size.

## Results

### Ethanol non-dependent (occasional drinkers) and ethanol dependent rats

Non-dependent rats, allowed to self-administer ethanol (10%) twice a week, self-administered an average dose of 1.21 ± 0.02 g/kg per session (30 min).

Daily ethanol consumption during chronic exposure to the alcoholic beverage (from 2.4 to 7.2% v/v ethanol) ranged from 6.30 ± 0.16 to 13.99 ± 0.66 g/kg. One-way ANOVA revealed a significant increase in ethanol consumption along the exposure time [*F*_(1, 52)_ = 127.18, *p* < 0.0001].

Average body weights in ethanol-dependent rats showed a progressive decrease from the beginning to the end of the study compared with ethanol non-dependent groups. However, significant main effects of group [*F*_(1, 33)_ = 2.57, *p* = 0.012], time [*F*_(1, 33)_ = 42.35, *p* < 0.0001] and a significant group x time interaction [*F*_(1, 33)_ = 62.21, *p* < 0.0001] revealed that the alcoholic beverage was responsible of a significant loss of body weight in ethanol-dependent rats.

### Somatic signs of withdrawal in dependent rats

**Figure 1:**
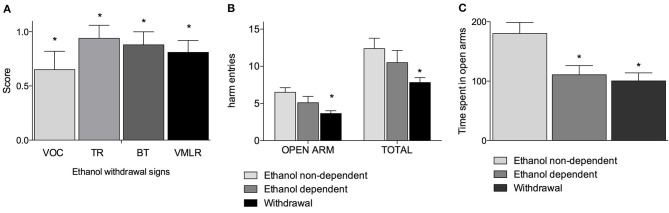
**Somatic signs of ethanol withdrawal assessed 12 h after the last ethanol intake in dependents rats**. Each withdrawal sign (VOC, TR, BT and VmLR) was assigned a score from 0 to 2. Values represent the mean (± s.e.m.) of 8–17 subjects per group. Ethanol non-dependent and ethanol dependent rats did not show withdrawal signs. Statistical difference with respect to the ethanol non-dependent (score: 0) and ethanol dependent (score: 0) groups (not shown in figure) was expressed as ^*^(A). Open arm entries and total arm entries were shown in **(B)**; time spent in open arms is shown in **(C)**. Values represent the mean (± s.e.m.) of 8–17 subjects per group. Statistical difference with respect to the ethanol non-dependent group was expressed as ^*^.

Figure 1A shows withdrawal signs, 12 h after suspension of the ethanol-enriched diet. Mann-Whitney *U*-tests, used to compare behavioral changes (scores) among ethanol withdrawal with respect to both ethanol non-dependent and ethanol-dependent groups, revealed a significant effect of withdrawal (*p* < 0.001). In particular, as shown in Figure 1A, analysis of individual withdrawal signs revealed a significant overall effect of ethanol withdrawal on VOC (*p* < 0.05), TL (*p* < 0.05), BT (*p* < 0.05) and in VmLR (*p* < 0.05) with respect to both ethanol non-dependent and ethanol-dependent groups. No changes were observed between ethanol non-dependent and ethanol-dependent groups.

Figures 1B,C shows the results of the anxiety-like behavior test following chronic exposure to ethanol on EPM test as determined by assessing the number of entries into open arms with respect to the total entries and average time spent into open arms. Repeated measures two way ANOVA revealed a significant main effect of treatment [*F*_(2, 32)_ = 4.78, *p* = 0.015], of open-closed/session entries [*F*_(1, 32)_ = 232.56, *p* < 0.0001] but not a significant treatment x open-closed/session entries interaction [*F*_(2, 32)_ = 2.62, *p* = 0.08] indicating a significant difference for entries into open arms between withdrawal vs. ethanol non-dependent group (*p* < 0.0001) and ethanol dependent group and a significant difference for entries into total arms between withdrawal vs. ethanol non-dependent group (*p* < 0.0001). In addition, there was a non-significant difference between withdrawal and ethanol-dependent group both into open arms and into total arms entries (Figure 1B). Moreover, one way ANOVA [*F*_(2, 32)_ = 11.86, *p* = 0.00014] indicated a significant difference for time spent into open arms between ethanol non-dependent vs. ethanol-dependent (*p* = 0.0015) and between ethanol non-dependent vs. withdrawal groups (*p* < 0.0001) (Figure 1C).

### Cystine/glutamate antiporter expression

**Figure 2:**
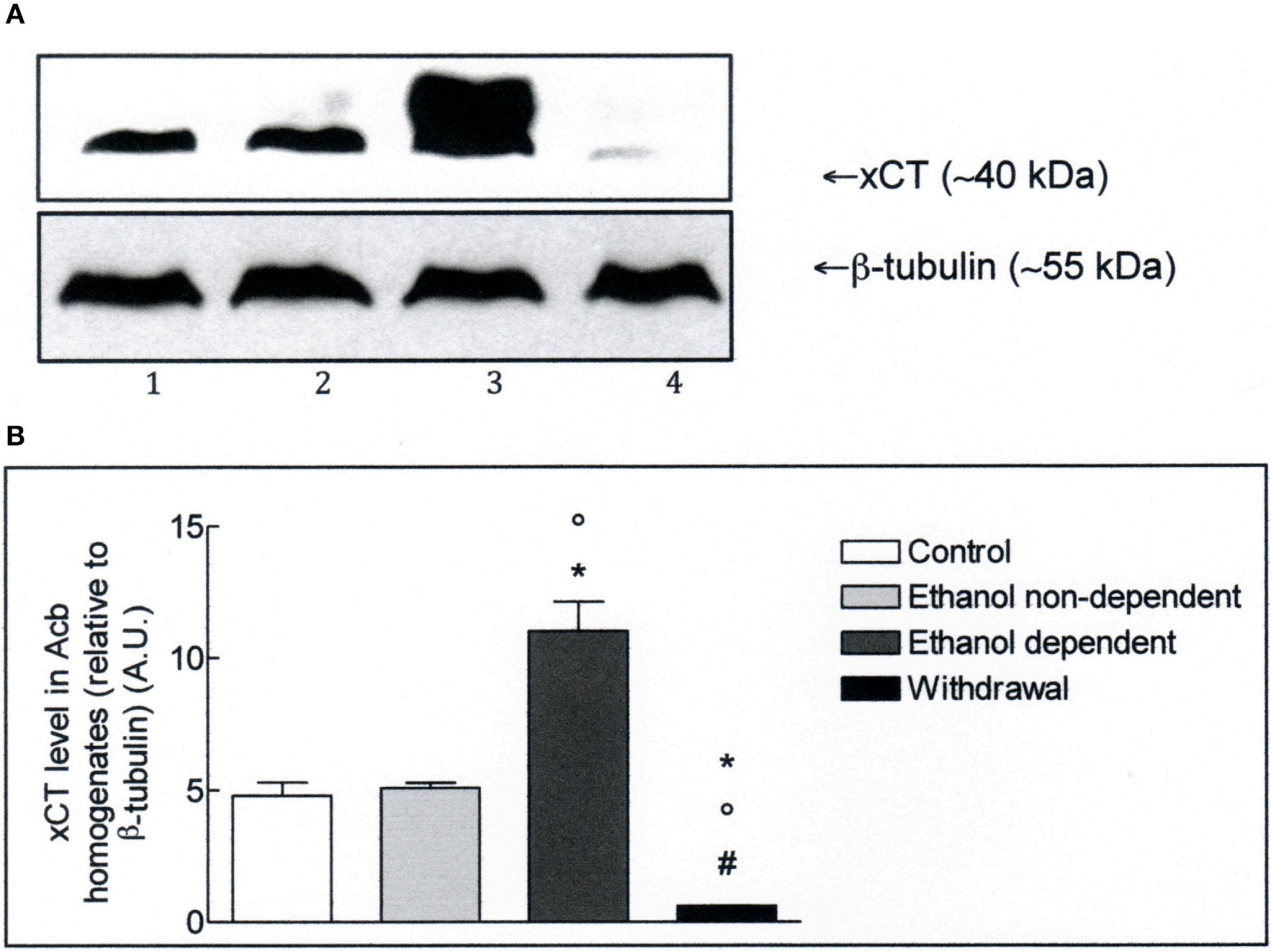
**(A)** xCT expression in Acb isolated from rat brain submitted to various treatments. The Acb homogenates were prepared as described in the Materials and Methods Section and processed by Western blot analysis using a specific polyclonal antibody against xCT. Lane 1: Control. Lane 2: ethanol non-dependent. Lane 3: ethanol-dependent. Lane 4: withdrawal. The bottom of the figure shows the level of β-tubulin in the same samples, used as control protein loading on the gel. The results showed are from one experiment out of three separate experiments. **(B)** Densitometric analysis of results showed in **(A)**. The level of xCT was normalized relative to β-tubulin (xCT/β-tubulin ratio × 10). Values represent the mean ± s.e.m. of three independent experiments and are expressed as arbitrary units (A.U.). ^*^p < 0.05 vs. control; °*p* < 0.05 vs. ethanol non-dependent; #*p* < 0.05 vs. ethanol-dependent rats.

The expression of xCT in Acb and whole brain homogenates was investigated by Western blot analysis. The antibody recognizes a band of about 40 kDa, which corresponds to the more active xCT isoform (Sato et al., 1999). Western blot experiments in lysates obtained from Acb (Figure 2A), revealed the presence of xCT in control group and a similar level of xCT expression in non-dependent rats. In ethanol-dependent rats there was a significant increase of xCT expression, compared to controls (*p* < 0.05) and non-dependent rats (*p* < 0.05). Following 12 h withdrawal in dependent rats, the level of xCT expression with respect to control (*p* < 0.05), non-dependent (*p* < 0.05) and ethanol-dependent (*p* < 0.05) samples, was significantly down regulated. The results of 3 experiments, expressed as A.U., are shown in Figure 2B.

**Figure 3:**
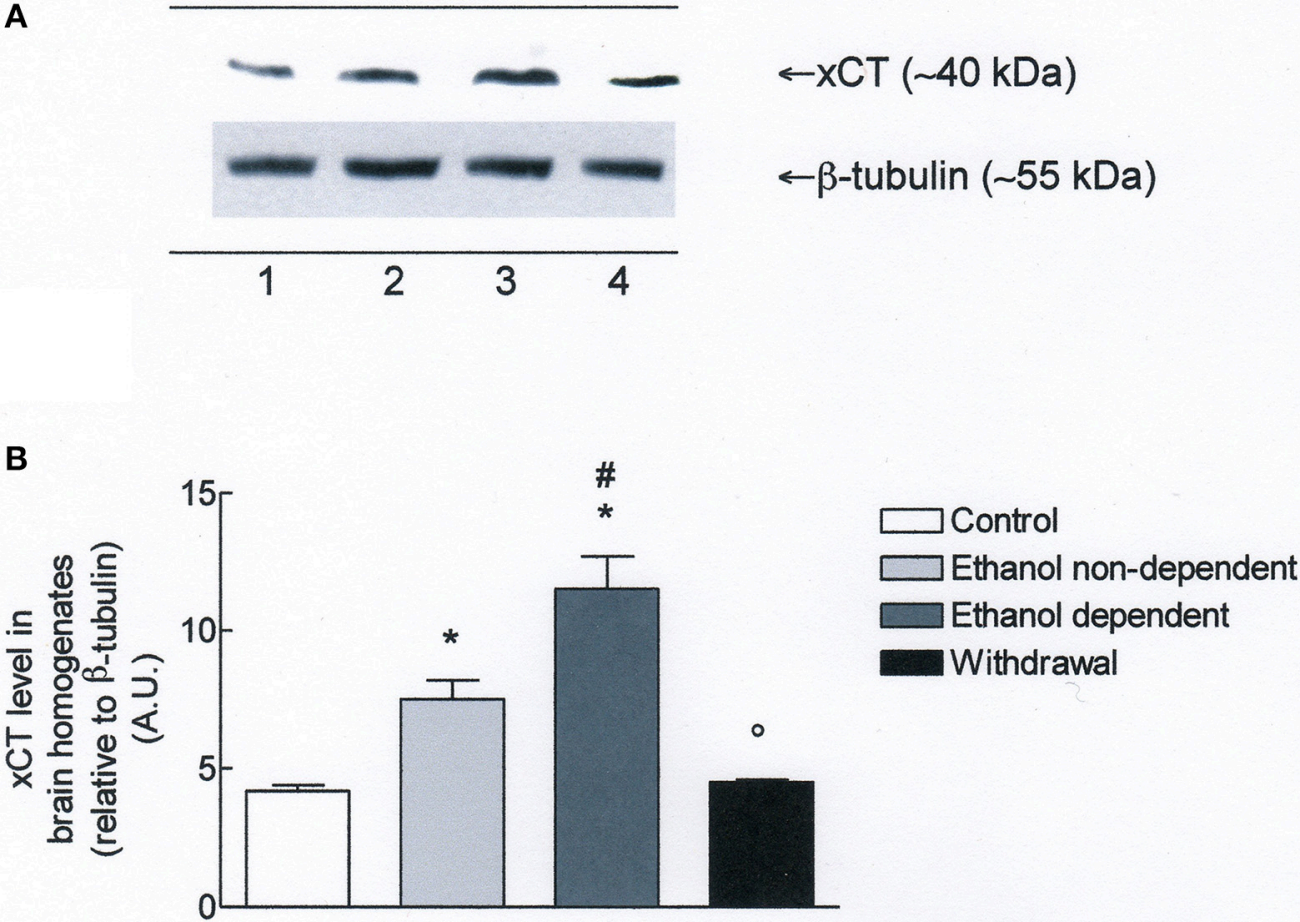
**(A)** xCT expression in rat brain homogenates. The brain homogenates were prepared as described in the Materials and Methods Section and processed by Western blot analysis using a specific polyclonal antibody against xCT. Lane 1: Control brain. Lane 2: ethanol non-dependent. Lane 3: ethanol-dependent. Lane 4: withdrawal. The bottom of the figure shows the level of β-tubulin in the same samples, used as control protein loading on the gel. The results showed are from a single experiment, repeated at least three times. **(B)** Densitometric analysis of results showed in **(A)**. The level of xCT was normalized relative to β-tubulin (xCT/β-tubulin ratio × 10). Values represent the mean ± s.e.m. of three independent experiments and are expressed as arbitrary units (A.U.). ^*^p < 0.05 vs. control; ^#^*p* < 0.05 vs. ethanol non-dependent °*p* < 0.05 vs. ethanol dependent rats.

Western blot experiments in lysates obtained from whole brain (Figure 3A), revealed the presence of xCT in control group. Moreover, xCT expression was significantly higher in ethanol non-dependent compared to control group (*p* < 0.05). The level of protein was even more increased in brain of rats dependent from ethanol, and this increase was significantly different from control group (*p* < 0.05) and ethanol non-dependent (*p* < 0.05). Following withdrawal, the level of xCT expression was restored to the level of control group (Figure 3B).

## Discussion

It is well known that many drugs of abuse can change the expression level of xCT in animal models of addiction and in humans (Knackstedt et al., 2009, 2010). The aim of the present study has been, therefore, to evaluate whether the expression of xCT may be modified by ethanol in the Acb and whole brain of ethanol non-dependent rats (occasional drinkers), ethanol-dependent rats, as well as, during ethanol withdrawal.

Strong evidence indicates that the disruption of glutamate homeostasis is associated with addictive disorders (Kalivas et al., 2009). The alterations in glutamate concentrations observed following prolonged exposure to drugs of abuse are associated with changes in the function and activity of several key components of the homeostatic control mechanism, including the xCT exchanger and the glial glutamate transporter (GLT-1). The glutamatergic system in the prefrontal cortex has been suggested to be involved in drug reinforcement (Goldstein and Volkow, 2002), and the role of glutamate projections from prefrontal cortex to the Acb and the VTA has been elucidated in clinical studies and in animal models of drug abuse (Goldstein and Volkow, 2002; Kalivas et al., 2009).

In the Acb core, about 60% of the basal level of extracellular glutamate is derived from the activity of the xCT (Baker et al., 2002). Many drugs of abuse can modify the glutamate concentration in the Acb, by interfering with the xCT activity or expression. The Acb is therefore regarded as a specialized brain area involved in the neurobiology of addiction (Reissner and Kalivas, 2010) and the xCT antiporter may be a target for the design of drugs to be used for the prevention of drug abuse (Bridges et al., 2012).

We hypothesized that similarly to the dependence mechanisms from other drugs of abuse, ethanol dependence may also change the expression of xCT. In our experiments, the xCT expression studied in the Acb homogenates from ethanol-dependent animals was strongly increased with respect to control group and ethanol non-dependent group (occasional drinkers). On the other hand, the xCT expression was strongly decreased in Acb homogenates of ethanol-dependent animals after 12 h withdrawal. These changes in xCT expression could be responsible for alterations in glutamate levels in the Acb that may occur in response to chronic ethanol exposure and that may contribute to the pathological glutamate signaling linked to withdrawal-induced behavior. The profound down-regulation in the expression of xCT in the Acb, following 12 h of withdrawal in dependents rats could be also linked to the recidivism for ethanol abuse after a period of abstinence (Knackstedt and Kalivas, 2009). The decrease of xCT expression in the Acb isolated from ethanol withdrawal group seems to be in line with other studies reporting similar results with two different addictive drugs, cocaine (Baker et al., 2003; Madayag et al., 2007; Kau et al., 2008; Knackstedt et al., 2010), and nicotine (Knackstedt et al., 2009), after extinction or after acute withdrawal of the drugs. It must be noted that, while chronic cocaine (Baker et al., 2003) and nicotine (Knackstedt et al., 2009), exposure may produce a decrease in basal extracellular glutamate levels (with reduced xCT expression possibly contributing to this effect), chronic ethanol exposure could result in an increased extracellular glutamate concentrations due to the xCT overexpression.

The balance between xCT and GLT-1 activities has a profound effect on the regulation of extrasynaptic glutamate levels and on the signaling through pre- and post-synaptic glutamate receptors, thus affecting synaptic plasticity and circuit-level activity (Kalivas et al., 2009). Besides the reduction of xCT expression, both cocaine and nicotine also reduce the levels of GLT-1 in the Acb, after extinction or after acute withdrawal of the drugs; moreover, the expression level of both xCT and GLT-1 was restored by the beta-lactam antibiotic ceftriaxone, known to induce the GLT-1 in the same experimental models (Knackstedt et al., 2009, 2010).

Other studies have demonstrated that ceftriaxone induces the up-regulation of the GLT-1 also in alcohol-preferring rats, and a dose-dependent reduction of ethanol consumption (Sari et al., 2011). Collectively, these data indicate that cocaine, nicotine, and ethanol could change the extracellular glutamate concentration in brain regions important for the development and/or maintenance of drug dependence via alteration of glutamate transporters. Our results suggest that ethanol dependence could increase the level of extracellular glutamate in the Acb, while ethanol withdrawal could decrease the amino acid concentration in the same brain area. This conclusion is based only on the xCT expression profile seen in the Acb and it is highly speculative, since we do not know what happens to the GLT-1 expression or to extracellular glutamate concentrations, which were not investigated in this work. However, many studies have demonstrated that the levels of extracellular glutamate are increased in central brain reward regions following chronic ethanol exposure and withdrawal (De Witte, 2004; Griffin Iii et al., 2014; Lum et al., 2014). If an increase in glutamate transmission plays a role in ethanol consumption associated with dependence, as these studies suggest, then the functional consequence of an increase/reduction of xCT exchanger in the Acb could be relevant in the development and/or manifestation of ethanol dependence (Knackstedt et al., 2009).

The results of Western blot analysis with whole brain homogenates showed that non-dependent rats (self-administered ethanol twice a week) have higher levels of xCT expression than control group, and a further significant increase of xCT in ethanol-dependent rats, in which xCT expression was reduced to control values after 12 h withdrawal. These data suggest that an intermittent (ethanol self-administration, twice a week), or a long-term ethanol exposure (in the liquid diet), cause an increase of the xCT in the whole brain. Interestingly, these results are in agreement with data obtained from Lin et al. (2013), who reported that ethanol treatment up-regulates xCT expression *in vitro* (Lin et al., 2013).

The activity of xCT contributes to the maintenance of a cellular redox balance, sufficient to protect cells from oxidative damage (Sato et al., 2005). Thus, the up-regulation of xCT expression might be a compensatory mechanism in response to ethanol-induced oxidative stress (Lin et al., 2013). Since the main function of xCT in the CNS, as well as in other systems, is to control the glutathione (GSH) synthesis, every condition that increases the production of oxygen free radicals, could lead to the up-regulation of xCT.

In many neurological conditions, such as inflammation/degenerative diseases (Pampliega et al., 2011), hypoxic diseases (Jackman et al., 2010), epilepsy and brain tumors (Lewerenz et al., 2014), the transcription of the *Slc7a11* gene is increased. In our opinion, the ethanol dependence can be considered similar to these pathological states, where there is a concomitant up-regulation of the xCT protein. It is noteworthy that ethanol metabolism generates reactive oxygen species and creates a state of oxidative stress in hepatocytes (Bailey et al., 1999). In response to oxidative stress, the transcription factor nuclear factor-erythroid 2-related factor 2 regulates expression of multiple genes involved in antioxidant defense mechanisms (McMahon et al., 2001). Moreover, oxygen and hydrogen peroxide are able to induce the xCT gene transcription in many experimental models (Sato et al., 2005). The increased expression of xCT caused by ethanol in the whole brain might be a general protective neuronal mechanism from oxidative stress, based on the increase of the GSH synthesis. In addition, the increased production of GSH, due to the higher activity of xCT, might also act as a link between antioxidant properties and the extracellular concentration of glutamate. As previously mentioned, the extracellular glutamate concentration is regulated by the xCT exchanger present mainly on glial cells, and by the high affinity Na^+^-dependent GLT-1, present on both glial and neuronal cells (Baker et al., 2002). Therefore, impaired function of these transporters can have a profound effect on extracellular glutamate concentrations and cause a dysregulation of glutamate homeostasis not only in the Acb, but also in the whole brain (Reissner and Kalivas, 2010). Nonetheless, studies in experimental animal models have shown that ethanol (Bailey et al., 1999; Dahchour et al., 2005), cocaine (Dietrich et al., 2005), heroin (Pan et al., 2005), and nicotine (Jain and Jaimes, 2013) increase reactive oxygen species.

The changes in xCT expression in Acb and whole brain induced by ethanol dependence seem to be tissue-specific, since we did not observe a similar pattern in rat spleen, an organ that shows a relatively high constitutive level of xCT expression (Taguchi et al., 2007) (data not shown).

In summary, our results show that ethanol dependence up-regulates the expression of xCT antiporter in the Acb, a structure involved in ethanol reinforcement, while 12 h ethanol withdrawal strongly reduces the expression of xCT. The down-regulation of xCT correlates with the withdrawal symptoms observed in animals and suggests that treatment with drugs able to restore the xCT level (i.e., N-acetylcysteine, ceftriaxone) could be useful for the management of ethanol withdrawal symptoms. xCT is also over-expressed in the whole brain homogenates from both non-dependent and ethanol dependent rats and restored to control level following ethanol withdrawal, probably as a consequence of the brain oxidative stress induced by ethanol exposure and by ethanol deprivation in withdrawal, respectively. Overall, these findings indicate that xCT expression is altered in both the Acb and the whole brain. xCT might therefore represent a novel therapeutic target for the treatment of ethanol abuse.

### Conflict of interest statement

The authors declare that the research was conducted in the absence of any commercial or financial relationships that could be construed as a potential conflict of interest.
